# Cardiometabolic Effects of Traditional South Asian Diets and the Growing Burden of Metabolic Syndrome

**DOI:** 10.26502/aimr.0248

**Published:** 2026-07-14

**Authors:** Akshay Bharadwaj, Meghana Kaipa, Devendra K. Agrawal

**Affiliations:** 1 Department of Translational Research, College of Osteopathic Medicine of the Pacific, Western University of Health Sciences, Pomona, California 91766 USA

**Keywords:** Cardiometabolic Disease, Chronic Inflammation, Dyslipidemia, Gut Dysbiosis, Insulin Resistance, Metabolic Syndrome, Oxidative Stress, South Asian Diet, Traditional Dietary Patterns, Visceral Adiposity

## Abstract

Metabolic syndrome is defined as a set of metabolic abnormalities that increase the risk of cardiovascular disease. South Asians are at a disproportionately high risk for metabolic syndrome due to their genetic profile as well as lifestyle and cultural factors. One main lifestyle factor adding to their increased prevalence is their dietary patterns, which include many high-risk food items like refined carbohydrates, oils, and saturated fats. These dietary components are interconnected with their cardiometabolic profiles which can lead to increasingly adverse outcomes across future South Asian generations. This paper evaluates the intricate relationship between metabolic syndrome and cardiovascular disease, specifically focusing on the dietary components that drive disease progression. Furthermore, this paper examines the protective cardiometabolic elements already inherent to the regional diet, exploring how traditional South Asian dietary patterns can be strategically modified to optimize preventive care. Ultimately, this review stresses the need for individually tailored, culturally centered dietary interventions to optimize preventive care and mitigate the metabolic burden in both native and diaspora South Asian communities.

## Introduction

Metabolic Syndrome (MetS) comprises a cluster of metabolic abnormalities that significantly elevate the risk of cardiovascular disease (CVD), such as myocardial infarction and peripheral vascular disease, type 2 diabetes mellitus, and complications in the intestinal, liver, and musculoskeletal diseases [1-14]. Uncontrolled diabetes and hyperlipidemia may destabilize atherosclerotic plaque in the carotid and coronary arteries [15-22], lead to steatohepatitis and fatty liver disease [7], peripheral arterial disease, diabetic foot ulcer [23], and musculoskeletal diseases [24], and may further accelerate the underlying pathogenesis of cardiometabolic syndrome. Many pharmaceutical agents, including second-generation anti-depressants may induce weight gain [25]. There is a complex interplay between stress to epithelial and endothelial cells, infections, immune signaling, and microbial factors supporting the role of inflammation due to metabolic syndrome and gut dysbiosis in the immune-metabolic interaction in cardiometabolic diseases [26-31]. It is indeed critical to effectively manage hyperglycemia and other risk factors of cardiometabolic syndrome [32-34].

Clinical diagnosis requires the presence of at least three diagnostic criteria, which include abdominal obesity (elevated waist circumference), impaired fasting glucose, and elevated systolic or diastolic blood pressure [1-4]. Globally, the prevalence of MetS continues to surge, affecting an estimated 1 billion individuals, with the number of cases doubling over the past two decades [35].

The South Asian region contributes disproportionately to this global burden, exhibiting some of the highest prevalence rates worldwide [35,36]. Consequently, South Asian populations face an accelerated risk of premature cardiometabolic morbidity. Many genetic factors play a role in this risk. Extensive research has shown that South Asian populations store less fat subcutaneously and more fat viscerally, contributing to abdominal obesity which is the primary driver of metabolic syndrome [37]. Additionally, there is marked insulin resistance and decreased pancreatic beta cell function in these populations, leading to a higher risk of type 2 diabetes which also contributes to MetS [37].

While genetic predispositions play a critical role, the high prevalence of MetS in South Asians is primarily driven by a dietary shift toward refined grains, fried foods, sugary beverages, and ultra-processed foods. Refined carbohydrates, such as white rice and all-purpose flour, alongside excessive dietary oil intake, and reduced protein intake are major culprits that exacerbate systemic inflammation, dyslipidemia, and insulin resistance, thereby predisposing South Asian individuals to MetS [38]. Conversely, distinct traditional components of the South Asian diet, most notably cardioprotective spices and fiber-rich lentils, offer substantial metabolic benefits [39].

However, translating these nutritional benefits into sustainable dietary changes is complicated by socioeconomic and cultural phenomena. Factors such as the high cost of fresh produce, increased working hours, and rapid adaptation to new cultures drive individuals toward low-quality, processed convenience foods rather than traditional, whole food items [40,41]. Furthermore, household gender dynamics and culinary traditions present challenges to individual dietary modification. This paper will evaluate the relationship between genetic susceptibility and dietary factors in driving South Asian MetS prevalence. Additionally, this review will examine the underlying socioeconomic and cultural barriers that hinder healthy eating behaviors, providing a framework for how traditional South Asian dietary patterns can be modified to serve as an effective preventative measure against cardiovascular disease.

## Methodology

The region of South Asia and the specific populations studied were defined according to world population databases. South Asia consists of the following nations: India, Pakistan, Bangladesh, Iran, Afghanistan, Nepal, Sri Lanka, Bhutan, and the Maldives. For the most part, this paper focused on the diets and practices of the three most populous nations of those listed - India, Bangladesh, and Pakistan [36]. This information was extracted from the Worldometer database using keywords like “South Asia” and “population” which resulted in data for each nation in the region. The data is split by year, spanning the years of 1955-2026, with the 2026 values being utilized for this paper.

The information in this article was gathered from sources published within the past 15 years found in the PubMed database (maintained by the National Library of Medicine). Using keywords such as “diet”, “insulin resistance”, “metabolic syndrome”, “ectopic fat”, and “gut dysbiosis”, relevant journal articles were found. These sources were especially helpful to understand the epidemiology and cardiometabolic characteristics of metabolic syndrome within South Asian populations, and which specific dietary components may lead to the diagnosis MetS.

## Epidemiology of Metabolic Syndrome

The escalating burden of MetS within South Asia exhibits distinct geographic, demographic, and generational patterns driven by the collision of genetic susceptibility with rapid environmental transitions. Regionally, the prevalence of MetS is significantly higher in urban centers than in rural or suburban areas [42]. This disparity is primarily driven by rapid urbanization, which introduces obesogenic elements, such as marked physical inactivity and energy-dense, ultra-processed diets, that severely exacerbate underlying genetic predispositions toward central adiposity and insulin resistance. Within these shifting populations, sex-specific differences further delineate risk; South Asian females experience a higher prevalence of MetS than their male counterparts, a vulnerability linked to elevated ectopic fat deposition and lower average skeletal muscle mass in women [42].

This gene-environment mismatch is even more pronounced among South Asian diaspora populations who migrate to Western nations, where the risk of type 2 diabetes mellitus (T2DM) and MetS escalates drastically relative to native cohorts. Data from the landmark Metabolic Syndrome and Atherosclerosis in South Asians Living in America (MASALA) study demonstrates that US-based South Asians experience a striking 23–27% prevalence of T2DM, compared to just 6–8% observed in non-Hispanic white cohorts [43]. While traditional regional diets possess inherent cardioprotective elements, diaspora populations frequently adopt distinct Western dietary risks like high-fat dairy products and high-sugar snacks, rapidly overwhelming their baseline metabolic tolerance [43].

On a longitudinal scale, tracking over the past three decades reveals a downward age shift in metabolic pathology. A particularly alarming trend is the manifestation of MetS risk factors within South Asian pediatric and adolescent populations, driven by physical inactivity early in childhood and the Westernization of youth diets [44]. When this early lifestyle shift meets a profound genetic predisposition for visceral adiposity and low lean mass, it results in premature metabolic programming, severely compromising the health of younger generations [45]. This localized public health crisis reflects a massive global macroeconomic shift; between 1990 and 2021, the global burden of diabetes- and hypertension-related mortality structurally displaced from high-income nations to low- and middle-income regions, positioning the South Asian subcontinent at the epicenter of the global cardiometabolic epidemic [46].

## Characteristics of Cardiometabolic Diseases

**Figure 1:**
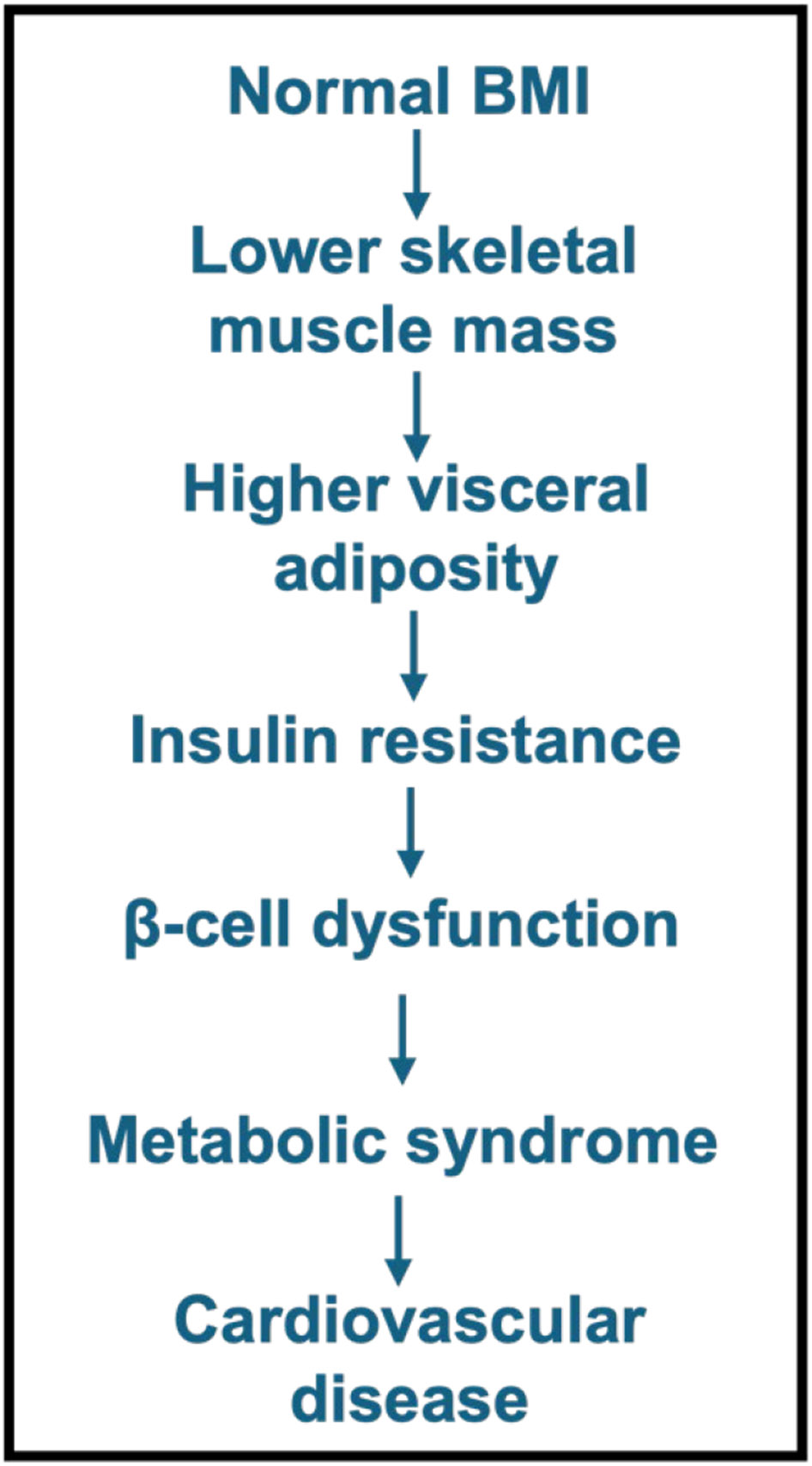
Metabolic Depiction of the South Asian “Thin Fat” Phenotype. The combination of elevated insulin and leptin levels, along with reduced skeletal muscle mass, contributes to the development of the “thin fat” phenotype commonly observed in South Asian populations [49]. This phenotype should be considered in routine screening and preventive health assessments, as individuals may present with a relatively lean overall appearance alongside increased central adiposity, which can be clinically misleading but is associated with a higher risk of metabolic syndrome.

Higher rates of obesity are prevalent among South Asians. Their phenotypes have been proven to display increased abdominal obesity at a lower BMI than White/Caucasian populations [47,48]. Even when BMI is slightly elevated, total and regional adiposity is elevated, which may be explained by metabolic hormones like leptin. Adipocytes directly secrete leptin as part of a negative feedback loop, and given the increased fat content in South Asians, leptin is produced much more significantly and is strongly associated with adiposity [48]. As displayed in Figure 1, the combination of higher insulin and leptin levels, indicating higher body fat, has been coined as the "thin-fat" phenotype [49]. This phenotype is considered a leading risk factor for MetS, and when combined with other factors such as lower skeletal muscle mass, South Asians are at much higher risk for the comorbidities associated with metabolic syndrome, including Type 2 Diabetes. Furthermore, the modernization and urbanization of the region reflect lifestyle changes noted in South Asians, including inadequate exercise patterns and increased consumption of refined carbohydrates and saturated fats [47,50].

Most cardiometabolic syndromes are interconnected, making it difficult to determine which condition develops first and subsequently contributes to another. Insulin resistance causes MetS and Type 2 Diabetes, both of which are highly prevalent in South Asian populations due to their diets and unique central fat distribution. However, this may represent a parallel mechanism wherein a higher propensity for ectopic hepatic fat accumulation and intramyocellular fat deposition contributes to insulin sensitivity abnormalities [51]. This, in turn, leads to impaired beta cell function and insulin action, increasing the likelihood of developing MetS and its co-existing conditions [49,51].

As a result of these interconnected metabolic and lifestyle factors, South Asians are more prone to dyslipidemia than European populations. This dyslipidemic profile is characterized by elevated LDL cholesterol, triglycerides, and lipoprotein(a), as well as decreased HDL cholesterol with more dysfunctional particles [52]. The accumulation of LDL cholesterol can contribute to atherosclerosis and hypertension, thereby increasing the likelihood of MetS. Additionally, these processes may lead to endothelial dysfunction, arterial stiffness, and premature vascular aging [53]. In contrast, HDL cholesterol serves a more protective role against atherosclerotic cardiovascular disease (ASCVD), and therefore lower levels are associated with an increased risk of cardiometabolic disorders [54].

Dietary patterns may further contribute to this cardiometabolic risk profile. Vegetarians make up 32.8% of the South Asian population, which has some benefits when they consume greater amounts of legumes, vegetables, root foods, and dairy, but it also means they may lack certain nutrients that are predominantly found in meat products, such as Vitamin B12 [49,50]. High folate levels and low B12 cause intrauterine undernutrition, leading to the development of a small, “thin fat” baby. If overnutrition occurs after birth, these individuals may subsequently develop obesity and hyperglycemia [49]. Additionally, vegetarians often consume more carbohydrates and less protein, leading to lower muscle mass compared with their non-vegetarian counterparts, which may place them at higher risk for MetS.

### Mechanisms Linking Dietary Components to Cardiometabolic Risk

South Asian diets are especially high in refined carbohydrates with most meals including some form of bread or rice, as depicted in Figure 3 [55-57]. Not only do these carbohydrate-rich foods make meals more filling, but they are also easy to incorporate and affordable, making them an integral part of dietary practices. Unfortunately, refined grain-based foods have a high glycemic load, causing impaired insulin signaling due to the rapid influx of glucose followed by increased insulin secretion to lower blood glucose levels. Over time, insulin receptors downregulate and decrease in sensitivity, resulting in insulin resistance (IR), impaired GLUT4 signaling, and hyperinsulinemia [58,59]. A notable marker of metabolic disorders, IR is commonly associated with Type 2 Diabetes Mellitus, MetS, Polyendocrine Metabolic Ovarian Syndrome (PMOS), cardiovascular disease, and metabolic dysfunction-associated steatotic liver disease (MASLD) [2-4,7,58].

Beyond their effects on insulin signaling, refined carbohydrates also contribute to systemic inflammation. A statistical analysis demonstrated a significant positive correlation between the inflammatory quality of diet, which is worsened by refined carbohydrate intake, and the increased incidence of cardiometabolic diseases [60]. Additionally, diets high in saturated fats and glucose promote the overproduction of reactive oxygen species (ROS), leading to oxidative stress [61]. This triggers inflammatory pathways that are modulated by factors such as NF-κB, cytokines, and CRP [62]. The oxidative stress and resulting chronic low-grade inflammation further alter the gut microbiome promoting the translocation of bacterial products which exacerbate systemic inflammation [61,63].

**Figure 2:**
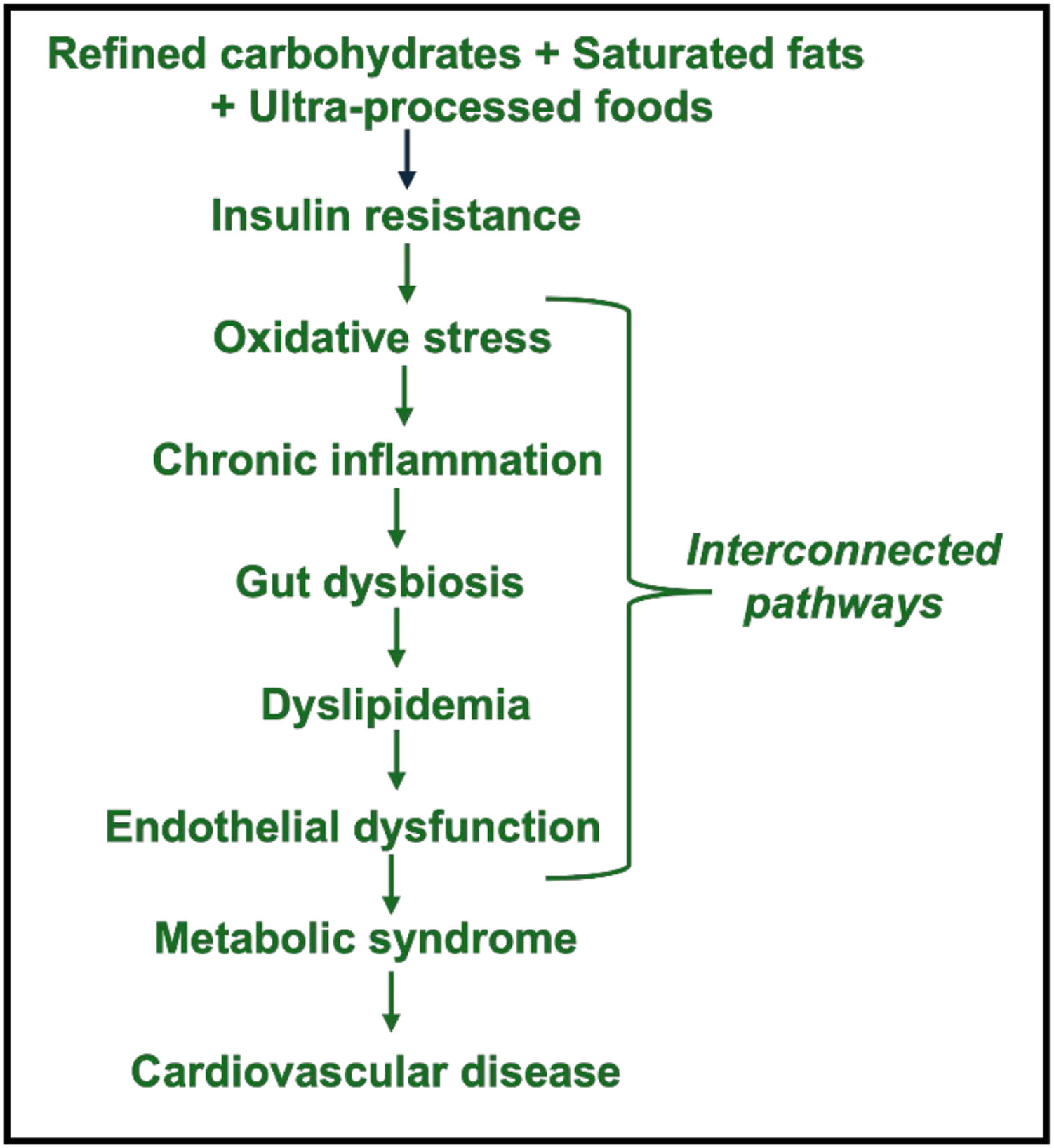
Mechanisms Linking Diet to Cardiometabolic Disease. Specific dietary components in South Asian diets, including refined carbohydrates, saturated fats, and ultra-processed foods, are associated with an increased risk of metabolic syndrome [58,59,60,61,63]. The mechanisms underlying this association are illustrated in the figure; however, it is important to note that these pathways are highly interconnected rather than strictly linear.

These inflammatory and microbiome alterations are accompanied by dyslipidemia and visceral adiposity, as these dietary patterns also promote ectopic fat accumulation in organs such as the liver, pancreas, and heart [58,59]. These metabolic disturbances are further influenced by the role of dietary fats in regulating cellular function through pathways involved in maintaining cardiometabolic health, such as gene transcription, mitochondrial function, membrane composition, and inflammatory signaling pathways [63]. Excess saturated fat intake results in endothelial dysfunction, which contributes to visceral adiposity and ectopic fat deposition [63]. As displayed in Figure 2, these pathways are highly interconnected, with oxidative stress, inflammation, gut microbiome alterations, and lipid dysregulation reinforcing one another in a feed-forward manner that amplifies metabolic dysfunction. Collectively, these metabolic disturbances increase overall cardiometabolic risk.

**Figure 3:**
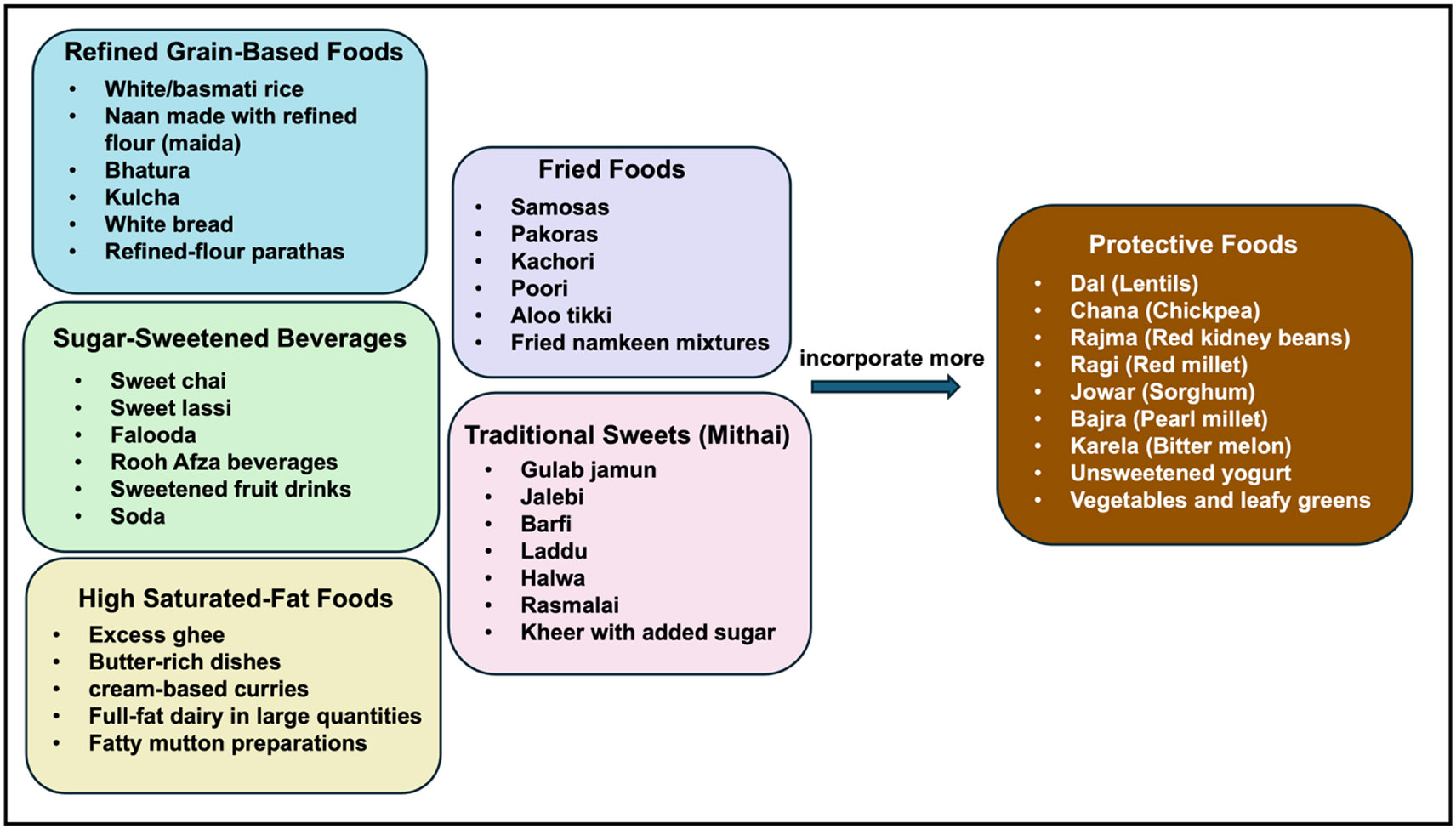
Examples of South Asian Foods Linked to Increased Cardiometabolic Risk. The foods/drinks/sweets listed in the different boxes are commonly used in both traditional and modern Asian diets [55-57]. Focusing on limiting or replacing items in these categories (as shown in the right box of protective foods) can help guide South Asian populations to create meals which are more protective against and reduce risks of developing cardiometabolic disorders.

To help lower this risk, these dietary patterns can be modified to better protect against cardiometabolic disorders. Certain foods, such as different types of lentils and spices (depicted in the brown box in Figure 3), have a lower glycemic index. By lowering the demand for insulin, digestion is slowed, allowing the compounds present in these foods with anti-inflammatory properties to exert beneficial effects. Modern diets should transition to incorporate a greater proportion of these foods, which may help prevent chronic inflammation, dyslipidemia, endothelial dysfunction, hyperinsulinemia, oxidative stress, and gut dysbiosis.

## Discussion

The typical elements of a South Asian diet should be looked at with respect to the unique genetic and cardiometabolic risk profile. Cardiometabolic multimorbidity (CMM), defined as the co-occurrence of two or more cardiometabolic conditions such as diabetes, ischemic heart disease, stroke, and other cardiovascular diseases, represents an increasingly important consequence of metabolic dysfunction in South Asian populations [64]. Dietary factors play a significant role in modulating this risk, as higher intakes of vitamin E, total fatty acids, polyunsaturated fatty acids, and monounsaturated fatty acids have been associated with a lower risk of CMM [64].

Conversely, several components of the traditional South Asian diet may contribute to cardiometabolic risk. Refined carbohydrates from white rice and excessive consumption of oil and sweets promote oxidative stress and metabolic dysfunction. The South Asian population’s genetic predisposition for lower skeletal muscle mass and decreased insulin sensitivity further exacerbates these effects, resulting in disproportionately exaggerated metabolic damage when compared to other ethnic groups [65]. However, protective elements are also prevalent within a South Asian diet. Lentils such as dal and chana provide high-fiber, low-glycemic loads that slow carbohydrate absorption and reduce insulin demands, while specific active compounds found in various spices exhibit anti-inflammatory properties that may help counteract the chronic systemic inflammation associated with MetS [2-4,66,67].

There are multiple socioeconomic and cultural barriers that limit adherence to a well-balanced diet. South Asian diaspora populations in Western nations are often faced with longer working hours and commute times, which steer them more towards cheap less nutritious foods. Oftentimes, it is difficult to convince older family members to make dietary changes because of tradition. South Asian meals have consisted of meals rich in oil and rice for many decades and many generations, and making this change, even gradually, can be difficult.

### Implications and Future Directions

South Asians may require a different approach to cardiometabolic risk assessment because traditional risk markers may underestimate disease burden. Through preventative measures such as clinical recognition of the "thin-fat" phenotype, earlier screening, and personalized dietary counseling, the prevalence of MetS among South Asians may be reduced.

Clinicians should recognize that South Asian patients may develop cardiometabolic abnormalities despite their relatively "thin" stature. Physicians may be accustomed to conventional BMI standards, where visually obese patients are considered at higher risk for MetS; however, this may not be the case for South Asian populations. Due to increased visceral adiposity, individuals with normal BMI values may still be at elevated cardiometabolic risk.

Screening for diabetes, metabolic syndrome, and cardiovascular disease at younger ages may also facilitate diagnosis at earlier stages of disease or identify patients with borderline values that place them at risk for future disease. Considering that adiposity is increased even at lower BMI values, lower thresholds for BMI and other parameters, such as waist circumference due to central obesity, should be considered when screening for MetS.

Rapid urbanization has accelerated the adoption of refined, energy-dense processed foods. While younger generations have been increasingly exposed to Western dietary patterns, dietary recommendations from clinicians often entail generic Western-style diets, such as the Mediterranean diet, which has demonstrated benefits against cardiometabolic conditions. However, these recommendations may not be effectively implemented because they differ substantially from traditional South Asian dietary practices. Instead, advising patients to reduce refined carbohydrates, deep-fried foods, and added sugars, while providing examples of commonly consumed foods within these categories (as depicted in Figure 3), may have a greater impact. Additionally, promoting traditional high-fiber and cardioprotective foods, such as vegetables, legumes, millets, dal, and chickpeas, could provide patients with a clearer understanding of what dietary changes are needed and how to implement them effectively. Promoting education through community nutrition programs, school-based interventions, public awareness campaigns, and preventative dietary counseling for individuals with a family history of cardiometabolic disease or associated risk factors may also help reduce disease prevalence.

## Conclusion

South Asians experience disproportionately high rates of metabolic syndrome and cardiovascular disease. This results from a combination of genetic susceptibility, and the transition to a more modern diet with increased refined carbohydrates, oils, and saturated fats. The urbanization of the region has rapidly modified both the diets and lifestyles (with inadequate exercise) of these populations. Traditional dietary practices contain both harmful and protective elements. Strategic and personalized modifications which preserve culturally important foods while reducing metabolically harmful components may substantially reduce cardiometabolic risk. To promote health outcomes, future prevention efforts should prioritize inventions specifically designed for South Asian populations, such as understanding South Asian phenotypes, personalizing dietary meal plans, promoting vitamin supplements that may be missing from current diets, and early screenings which keep in mind lower thresholds for BMIs and other metrics used to diagnose cardiometabolic diseases.

The success of these prevention techniques relies on accounting for the socioeconomic realities of food access and costs, alongside the deeply ingrained familial structures that govern South Asian households. Because dietary habits are rarely individual choices in this community, interventions must shift away from standard primary care mandates toward inclusive, family-centered models of care. As the global burden of metabolic disease continues to concentrate within South Asian populations, implementing these strategies is an urgent imperative required to dismantle this profound gene-environment mismatch and safeguard the metabolic longevity of future generations.

## Key Points

- Metabolic syndrome and cardiovascular disease are disproportionately prevalent in South Asian populations due to the interaction between genetic susceptibility and rapid dietary and lifestyle transitions.
- A unique cardiometabolic phenotype characterized by increased visceral adiposity, insulin resistance, decreased pancreatic β-cell function, and lower skeletal muscle mass contributes to elevated metabolic risk, even at lower BMI values.
- The "thin fat" phenotype, characterized by increased body fat despite a relatively lean appearance, is a major contributor to the development of metabolic syndrome and Type 2 Diabetes.
- Rapid urbanization, westernization of dietary patterns, and physical inactivity have accelerated the prevalence of metabolic syndrome across both native and diaspora populations.
- Traditional dietary patterns high in refined carbohydrates, saturated fats, excessive oils, and ultra-processed foods contribute to insulin resistance, chronic inflammation, oxidative stress, dyslipidemia, and endothelial dysfunction.
- These metabolic disturbances promote ectopic fat accumulation, gut microbiome alterations, and a feed-forward cycle of cardiometabolic dysfunction that increases the risk of cardiovascular disease and cardiometabolic multimorbidity.
- A characteristic dyslipidemia profile consisting of elevated triglycerides, LDL cholesterol, and lipoprotein(a), together with decreased HDL cholesterol, further increases cardiovascular risk.
- Protective dietary components, including lentils, legumes, millets, vegetables, and bioactive compounds found in spices, may improve glycemic control, reduce inflammation, and lower cardiometabolic risk.
- Traditional cardiometabolic risk assessments may underestimate disease burden; therefore, earlier screening, recognition of the "thin fat" phenotype, and lower BMI and waist circumference thresholds should be considered.
- Personalized and culturally tailored dietary modifications that preserve traditional protective foods while reducing metabolically harmful dietary components may substantially reduce the burden of metabolic syndrome and cardiovascular disease.

## References

[1] Swarup S, Ahmed I, Grigorova Y, Zeltser R. Metabolic Syndrome. 2024 Mar 7. In: StatPearls [Internet]. Treasure Island (FL): StatPearls Publishing; 2026 Jan–. 29083742

[2] Satish M, Saxena SK, Agrawal DK. Adipokine Dysregulation and Insulin Resistance with Atherosclerotic Vascular Disease: Metabolic Syndrome or Independent Sequelae? J Cardiovasc Transl Res. 12 (2019):415–424. 30835048 10.1007/s12265-019-09879-0 PMC6728239

[3] Patel S, Patel H, Mukadam S, Agrawal DK. Apolipoprotein B in the Risk Assessment, Diagnosis, and Treatment of Cardiometabolic Diseases. Cardiol Cardiovasc Med 9 (2025): 427–438. 41346943 10.26502/fccm.92920466 PMC12674602

[4] Patial R, Batta I, Thakur M, Sobti RC, Agrawal DK. Etiology, Pathophysiology, and Treatment Strategies in the Prevention and Management of Metabolic Syndrome. Arch Intern Med Res 7 (2024): 273–283. 39574946 10.26502/aimr.0184 PMC11580789

[5] Tang XL, Alloosh M, Ou Q, A new model of heart failure with preserved ejection fraction induced by metabolic syndrome in Ossabaw miniature swine. Basic Res Cardiol 120 (2025): 559–578. 40312575 10.1007/s00395-025-01112-1 PMC12159109

[6] Pelham CJ, Drews EM, Agrawal DK. Vitamin D controls resistance artery function through regulation of perivascular adipose tissue hypoxia and inflammation. J Mol Cell Cardiol 98 (2016): 1–10. 27374117 10.1016/j.yjmcc.2016.06.067 PMC5026596

[7] Radwan MM, Radwan BM, Nandipati KC, Hunter WJ 3rd, Agrawal DK. Immunological and molecular basis of nonalcoholic steatohepatitis and nonalcoholic fatty liver disease. Expert Rev Clin Immunol. 9 (2013): 727–738. 23971751 10.1586/1744666X.2013.816484

[8] Swier VJ, Tang L, Radwan MM, Hunter WJ 3rd, Agrawal DK. The role of high cholesterol-high fructose diet on coronary arteriosclerosis. Histol Histopathol 31 (2016): 167–176. 26260796 10.14670/HH-11-652

[9] Jia G, Cheng G, Gangahar DM, Agrawal DK. Involvement of connexin 43 in angiotensin II-induced migration and proliferation of saphenous vein smooth muscle cells via the MAPK-AP-1 signaling pathway. J Mol Cell Cardiol 44 (2008): 882–890. 18405916 10.1016/j.yjmcc.2008.03.002 PMC2765202

[10] Kandikattu S, Aavula M, Pisarski T, Parvizi D, Agrawal DK. Effect of Metabolic Syndrome in the incidence of Rotator Cuff Injury and Recovery following Surgical Repair. J Surg Res (Houst). 8 (2025): 202–210. 40519247 10.26502/jsr.10020438 PMC12165462

[11] Raney EB, Thankam FG, Dilisio MF, Agrawal DK. Pain and the pathogenesis of biceps tendinopathy. Am J Transl Res 9 (2017): 2668–2683. 28670360 PMC5489872

[12] Makinde T, Murphy RF, Agrawal DK. Immunomodulatory role of vascular endothelial growth factor and angiopoietin-1 in airway remodeling. Curr Mol Med. 6 (2006): 831–841. 17168735 10.2174/156652406779010795

[13] Makinde T, Agrawal DK. Intra and extravascular transmembrane signalling of angiopoietin-1-Tie2 receptor in health and disease. J Cell Mol Med. 12 (2008): 810–828. 18266978 10.1111/j.1582-4934.2008.00254.x PMC4401129

[14] Hall SC, Fischer KD, Agrawal DK. The impact of vitamin D on asthmatic human airway smooth muscle. Expert Rev Respir Med 10 (2016): 127–135. 26634624 10.1586/17476348.2016.1128326 PMC4955853

[15] Velpuri P, Patel P, Yazdani A, Abdi A, Rai V, Agrawal DK. Increased Oxidative Stress and Decreased Sirtuin-3 and FOXO3 Expression Following Carotid Artery Intimal Injury in Hyperlipidemic Yucatan Microswine. Cardiol Cardiovasc Med 8 (2024): 33–42. 38333571 10.26502/fccm.92920355 PMC10852345

[16] Truong R, Thankam FG, Agrawal DK. Immunological mechanisms underlying sterile inflammation in the pathogenesis of atherosclerosis: potential sites for intervention. Expert Rev Clin Immunol 17 (2021): 37–50. 33280442 10.1080/1744666X.2020.1860757 PMC7906938

[17] Velpuri P, Rai V, Agrawal DK. Role of sirtuins in attenuating plaque vulnerability in atherosclerosis. Mol Cell Biochem. 479 (2024): 51–62. 36952068 10.1007/s11010-023-04714-2 PMC10034899

[18] Jia G, Cheng G, Gangahar DM, Agrawal DK. Insulin-like growth factor-1 and TNF-alpha regulate autophagy through c-jun N-terminal kinase and Akt pathways in human atherosclerotic vascular smooth cells. Immunol Cell Biol. 84 (2006): 448–454. 16942488 10.1111/j.1440-1711.2006.01454.x

[19] Noothi SK, Ahmed MR, Agrawal DK. Residual risks and evolving atherosclerotic plaques. Mol Cell Biochem 478 (2023): 2629–2643. 36897542 10.1007/s11010-023-04689-0 PMC10627922

[20] Rai V, Rao VH, Shao Z, Agrawal DK. Dendritic Cells Expressing Triggering Receptor Expressed on Myeloid Cells-1 Correlate with Plaque Stability in Symptomatic and Asymptomatic Patients with Carotid Stenosis. PLoS One. 11 (2016): e0154802. 27148736 10.1371/journal.pone.0154802 PMC4858252

[21] Rao VH, Rai V, Stoupa S, Subramanian S, Agrawal DK. Tumor necrosis factor-α regulates triggering receptor expressed on myeloid cells-1-dependent matrix metalloproteinases in the carotid plaques of symptomatic patients with carotid stenosis. Atherosclerosis. 248 (2016): 160–169. 27017522 10.1016/j.atherosclerosis.2016.03.021 PMC4836960

[22] Rao VH, Rai V, Stoupa S, Agrawal DK. Blockade of Ets-1 attenuates epidermal growth factor-dependent collagen loss in human carotid plaque smooth muscle cells. Am J Physiol Heart Circ Physiol 309 (2015): H1075–1086. 26254334 10.1152/ajpheart.00378.2015 PMC4591361

[23] Shofler D, Rai V, Mansager S, Cramer K, Agrawal DK. Impact of resolvin mediators in the immunopathology of diabetes and wound healing. Expert Rev Clin Immunol 17 (2021): 681–690. 33793355 10.1080/1744666X.2021.1912598

[24] Fang WH, Bonavida V, Agrawal DK, Thankam FG. Hyperlipidemia in tendon injury: chronicles of low-density lipoproteins. Cell Tissue Res. 392 (2023): 431–442. 36738312 10.1007/s00441-023-03748-8 PMC10172214

[25] Mouawad M, Nabipur L, Agrawal DK. Impact of Antidepressants on Weight Gain: Underlying Mechanisms and Mitigation Strategies. Arch Clin Biomed Res 9 (2025): 183–195. 40444017 PMC12121960

[26] Perry S, Pillarisetti L, Gelfman T, Agrawal DK. Gut-Brain Axis in Inflammatory Bowel Disease: Pathogenesis and Therapeutics. Arch Intern Med Res 8 (2025): 339–345. 41568320 10.26502/aimr.0227 PMC12818948

[27] Maličević U, Rai V, Skrbic R, Agrawal DK. Hyperglycemia impairs the expression of inflammatory mediators in rat intestine: an implication for intestinal inflammation and inflammatory bowel disease. Mol Cell Biochem. 2026 Mar;481(3):1325–1337. 41511724 10.1007/s11010-025-05474-x PMC12995948

[28] Stojanovic M, Mendoza-Mari Y, Rai V, Agrawal DK. Hyperglycemia alters the gene and protein expression of CDC42 in small and large intestine of Sprague-Dawley rats. Mol Cell Biochem. 481 (2026): 1279–1291. 41511725 10.1007/s11010-025-05479-6 PMC12996419

[29] Pathak A, Agrawal DK. Role of Gut Microbiota in Long COVID: Impact on Immune Function and Organ System Health. Arch Microbiol Immunol 9 (2025): 38–53. 40051430 PMC11883900

[30] Malicevic U, Rai V, Skrbic R, Agrawal DK. NLRP3 Inflammasome and Gut Dysbiosis Linking Diabetes Mellitus and Inflammatory Bowel Disease. Arch Intern Med Res 7 (2024): 200–218. 39328924 10.26502/aimr.0178 PMC11426418

[31] Malicevic U, Rai V, Skrbic R, Agrawal DK. Intricate interplay between ORMDL3, ER stress, and autophagy in the diabetic intestine. Mol Cell Biochem. 481 (2026): 791–807. 41182648 10.1007/s11010-025-05424-7 PMC12963212

[32] Pillarisetti L, Agrawal DK. Semaglutide: Double-edged Sword with Risks and Benefits. Arch Intern Med Res 8 (2025): 1–13. 39902055 10.26502/aimr.0189 PMC11790292

[33] Vayser D, Wang P, Dafesh N, Agrawal DK. Outcomes of Diabetes Management with Continuous Glucose Monitoring Technology. Arch Intern Med Res 9 (2026): 119–135. 42306173 10.26502/aimr.0243 PMC13268403

[34] Radparvar I, Agrawal DK. A Critical Analysis of the Clinical Use of Incretin-Based Therapies: Efficacy and Adverse Events. Arch Clin Med Case Rep 9 (2025): 242–253. 41835241 10.26502/acmcr.96550740 PMC12981728

[35] Noubiap JJ, Nansseu JR, Nyaga UF. Worldwide trends in metabolic syndrome from 2000 to 2023: a systematic review and modelling analysis. Nat Commun 17 (2026): 573. 10.1038/s41467-025-67268-5 PMC12808104 41350289

[36] Population of Southern Asia (2026) - Worldometer [Internet]. [cited 2026 Jun.19]. https://www.worldometers.info/world-population/southern-asia-population/.

[37] Hodgson S, Williamson A, Bigossi M. Genetic basis of early onset and progression of type 2 diabetes in South Asians. Nat Med 31 (2025): 323–331. 39592779 10.1038/s41591-024-03317-8 PMC11750703

[38] Anjana RM, Sudha V, Abirami K. Dietary profiles and associated metabolic risk factors in India from the ICMR–INDIAB survey-21. Nat Med 31 (2025): 3813–3824. 41028544 10.1038/s41591-025-03949-4 PMC12618255

[39] Gupta Jain S, Puri S, Misra A, Gulati S, Mani K. Effect of oral cinnamon intervention on metabolic profile and body composition of Asian Indians with metabolic syndrome: a randomized double -blind control trial. Lipids Health Dis. 16 (2017): 113. 28606084 10.1186/s12944-017-0504-8 PMC5469078

[40] Russell C, Whelan J, Love P. Assessing the Cost of Healthy and Unhealthy Diets: A Systematic Review of Methods. Curr Nutr Rep 11 (2022): 600–617. 36083573 10.1007/s13668-022-00428-x PMC9461400

[41] Gurumurthy G, Agrawal DK. Impact of Maternal Ultra-Processed Food Consumption and Preterm Birth on the Development of Metabolic Disorders in Offspring. J Pediatr Perinatol Child Health 9 (2025): 68–84. 40519768 10.26502/jppch.74050214 PMC12165461

[42] Krishnamoorthy Y, Rajaa S, Murali S, Rehman T, Sahoo J, Prevalence of metabolic syndrome among adult population in India: A systematic review and meta-analysis. PLOS ONE 15 (2020): e0240971. 33075086 10.1371/journal.pone.0240971 PMC7571716

[43] Gujral UP, Kanaya AM. Epidemiology of diabetes among South Asians in the United States: lessons from the MASALA study. Ann N Y Acad Sci 1495 (2021): 24–39. 33216378 10.1111/nyas.14530 PMC8134616

[44] Wolf RM, Nagpal M, Magge SN. Diabetes and cardiometabolic risk in South Asian youth: A review. Pediatr Diabetes 22 (2021): 52–66. 32666595 10.1111/pedi.13078 PMC8191592

[45] Rajamoorthi A, LeDuc CA, Thaker VV. The metabolic conditioning of obesity: A review of the pathogenesis of obesity and the epigenetic pathways that "program" obesity from conception. Front Endocrinol (Lausanne) 13 (2022): 1032491. 36329895 10.3389/fendo.2022.1032491 PMC9622759

[46] Khanna S, Gan GCH, Sindone AP, Asia at the Epicenter of the Global Cardiometabolic Shift. JACC Asia 6 (2026): 269–283. 41778695 10.1016/j.jacasi.2026.01.001 PMC12959306

[47] Misra A, Soares MJ, Mohan V, Body fat, metabolic syndrome and hyperglycemia in South Asians. J Diabetes Complications 32 (2018): 1068–1075. 30115487 10.1016/j.jdiacomp.2018.08.001

[48] Shah A, Hernandez A, Mathur D, Adipokines and body fat composition in South Asians: results of the Metabolic Syndrome and Atherosclerosis in South Asians Living in America (MASALA) study. Int J Obes (Lond) 36 (2012): 810–816. 21863003 10.1038/ijo.2011.167 PMC3224670

[49] Mohan V. Lessons learned from epidemiology of type 2 diabetes in South Asians: Kelly West Award Lecture 2024. Diabetes Care 48 (2025): 153–163. 39841965 10.2337/dci24-0046 PMC11770170

[50] Shridhar K, Dhillon PK, Bowen L, Nutritional profile of Indian vegetarian diets--the Indian Migration Study (IMS). Nutr J. 13 (2014): 55. 24899080 10.1186/1475-2891-13-55 PMC4055802

[51] Narayan KMV, Kanaya AM. Why are South Asians prone to type 2 diabetes? A hypothesis based on underexplored pathways. Diabetologia 63 (2020): 1103–1109. 32236731 10.1007/s00125-020-05132-5 PMC7531132

[52] Saeed A, Virani SS, Mulukutla S, Chow CK. Dyslipidemia and Cardiovascular Disease Prevention in South Asians: A Review and Discussion of Causes, Challenges and Management Strategies. Curr Diabetes Rev 17 (2021): e011221190238. 33438542 10.2174/1573399817999210112192419

[53] Gholap N, Davies M, Patel K, Sattar N, Khunti K. Type 2 diabetes and cardiovascular disease in South Asians. Prim Care Diabetes 5 (2011): 45–56. 20869934 10.1016/j.pcd.2010.08.002

[54] Razavi AC, Jain V, Grandhi GR, Does Elevated High-Density Lipoprotein Cholesterol Protect Against Cardiovascular Disease? J Clin Endocrinol Metab 109 (2024): 321–332. 37437107 10.1210/clinem/dgad406 PMC11032254

[55] Radhika G, Sathya RM, Ganesan A, Dietary profile of urban adult population in South India in the context of chronic disease epidemiology (CURES-68). Public Health Nutr. 14 (2011): 591–598. 20701818 10.1017/S136898001000203X

[56] Prabhakaran D, Kapur K, Graubard BI, A cross-sectional investigation of regional patterns of diet and cardio-metabolic risk in India. Nutr J 10 (2011): 12. 21276235 10.1186/1475-2891-10-12 PMC3042918

[57] Misra A, Singhal N, Sivakumar B, Bhagat N, Jaiswal A, Khurana L. Nutrition transition in India: secular trends in dietary intake and their relationship to diet-related non-communicable diseases. J Diabetes 3 (2011): 278–292. 21649865 10.1111/j.1753-0407.2011.00139.x

[58] Caretto A, Zanardini A, Frontino G, Pedone E. Targeting Insulin Resistance Through Nutrition: Pathophysiological Insights and Dietary Interventions. Nutrients 18 (2026): 1119. 41978168 10.3390/nu18071119 PMC13074555

[59] Pappu A, Mishra N, Mishra S. Culturally Informed Dietary Approaches for Cardiometabolic Risk Reduction in South Asians: An Evidence-Based Review. J Clin Med 15 (2026): 1421. 41753110 10.3390/jcm15041421 PMC12942075

[60] Aslani Z, Sadeghi O, Heidari-Beni M, Association of dietary inflammatory potential with cardiometabolic risk factors and diseases: a systematic review and dose-response meta-analysis of observational studies. Diabetol Metab Syndr 12 (2020): 86. Erratum in: Diabetol Metab Syndr 12 (2020): 106. 33117453 10.1186/s13098-020-00592-6 PMC7590706

[61] Sverdlov AL, Figtree GA, Horowitz JD, Ngo DT. Interplay between Oxidative Stress and Inflammation in Cardiometabolic Syndrome. Mediators Inflamm. (2016): 8254590. 27563174 10.1155/2016/8254590 PMC4985569

[62] Biobaku Fatimo, Ghanim Husam, Batra Manav, Dandona Paresh, Macronutrient-Mediated Inflammation and Oxidative Stress: Relevance to Insulin Resistance, Obesity, and Atherogenesis, The Journal of Clinical Endocrinology & Metabolism 104 (2019): 6118–6128. 31219543 10.1210/jc.2018-01833

[63] Carías Domínguez AM, de Jesús Rosa Salazar D, Stefanolo JP, Cruz Serrano MC, Casas IC, Zuluaga Peña JR. Intestinal Dysbiosis: Exploring Definition, Associated Symptoms, and Perspectives for a Comprehensive Understanding - a Scoping Review. Probiotics Antimicrob Proteins 17 (2025): 440–449. 39235661 10.1007/s12602-024-10353-w PMC11832579

[64] Zhang Y, Cen Y, Li X, Yang B, Xing K, Lian L, Yu Y, Zhao Y. Vitamin E and Fatty Acid Intake and Cardiometabolic Multimorbidity Risk: The Mediating Role of Plasma Lipid Metabolites. Int J Mol Sci 26 (2025): 11477. 41373632 10.3390/ijms262311477 PMC12691755

[65] Bhardwaj B, O'Keefe EL, O'Keefe JH. Death by Carbs: Added Sugars and Refined Carbohydrates Cause Diabetes and Cardiovascular Disease in Asian Indians. Mol Med 113 (2016): 395–400. PMC6139832 30228507

[66] Clarke ST, Sarfaraz S, Qi X, Ramdath DG, Fougere GC, Ramdath DD. A Review of the Relationship between Lentil Serving and Acute Postprandial Blood Glucose Response: Effects of Dietary Fibre, Protein and Carbohydrates. Nutrients 14 (2022): 849. 35215500 10.3390/nu14040849 PMC8877848

[67] Alkhatib DH, Jaleel A, Tariq MNM, The Role of Bioactive Compounds from Dietary Spices in the Management of Metabolic Syndrome: An Overview. Nutrients. 2021; 14 (2021): 175. 35011050 10.3390/nu14010175 PMC8747161

